# Positive medical education: Are we focusing on the right things while teaching?

**DOI:** 10.15694/mep.2018.0000156.1

**Published:** 2018-07-26

**Authors:** Alvaro Tala

**Affiliations:** 1 Universidad de Chile

**Keywords:** Positive Psychology, Positive Education, Medical Education, Well-being

## Abstract

This article was migrated. The article was marked as recommended.

Neuropsychiatric disorders are a global problem and medical students are a population with high vulnerability to mental disorders, with medical education currently in crisis considering the alarming rates of mental health problems reported in medical students, which in addition to compromising the health of students, influence their learning processes and patient care. In this context, it is necessary to move towards a medical education that considers, in addition to the academic performance, the well-being of its students as a central focus, without compromising the former. Positive psychology, with interventions at individual level, and positive education, with interventions at institutional level, could provide a response based on the evidence to this problem, in the form of a positive medical education. This article address a personal view of how this approach can benefit medical students

## Perspective

From my point of view, medical education and well-being have a close relationship in at least two points: on one hand, how the teaching and learning processes are developed can influence the well-being of the students, with studies that have showed that formative processes in general tend to generate stress in the students, associating this to greater rates of adverse outcomes in mental health, being medical students a population with greater vulnerability to mental disorders compared to populations of similar age range, due to factors such as role transition, sleep deprivation, poor support systems, among others, with studies that have shown rates of depressive symptoms up to almost 30% and suicidal ideation of up to 11% in this population ( Rotenstein *et al.*, 2016), and given how the learning environments are structured nowadays, medical students tend to neglect their well-being in order to prioritize their academic performance, this despite the efforts of multiple universities around the world. Although universities have implemented various strategies to promote the well-being of their students from different approaches, they have not been able to find a definitive answer to this problem, and even many times are being confronted with the fact that students do not value these strategies as useful for their well-being.

On the other hand, there is compelling evidence that associates elements of well-being not only with better results in physical health, but also mental health, not explained only by the absence of disease, and that these elements of well-being could also be associated with better learning outcomes, higher achievement, and greater labor productivity, possibly through mechanisms such as increased creativity, cognitive flexibility, holistic thinking,resilience, intrinsic motivation among others ( Seligman *et al.*, 2009; Diener *et al.*, 2017; Myers and Diener, 2018).

In this context, I think the work done by positive psychology in the topic of well-bieng and education stands out. Under the umbrela of positive psychology exists positive education, which is an approach to education that blends academic learning with well-being. Within this approach, many great scientists in fields like psychology and neurosciences, like Martin Seligman, have managed to carry out initiatives worldwide, in countries like United Kingdom, Australia, Mexico and United States of America, both in schools and universities, in order to generate concrete educational programs, with measurable results, on the basis that well-being would have aspects that could be intervened and strengthened. To the moment, interventions have shown that they could prevent adverse results in mental health like depression, at least in young people, and that they can increase good relationships, engagement in learning, cooperation, assertiveness, and self-control, among other qualities that I think can benefit medical students to meet the needs of the modern healthcare, but in spite of that, maybe due to the need of gather more data before include this in medical education to avoid potential harms to students and patients, there is a lack of data related to positive education in medical curriculums across the world. In my opinion, more important than the specific interventions, which still require further studies to ensure their effectiveness, is the contribution that the paradigm of positive psychology offers to medical education in the question: “what is the purpose of the teaching/learning?”. From my point of view, the ultimate answer to this question could be that we learn and teach in order to maximize the happiness or well-being of individuals and eventually of society, being the educational institutions a fundamental place from which this subject can be worked, since they are environments that frequently affect the well-being of students, and since they are the place where students spend most of their time. Although the World Health Organization (WHO) defines health not only as the absence of disease, but as a state of complete physical, mental and social well-being, in my experience from medical education, and particularly in mental health, the time students spend in medical education tends to focuses particularly on disorders and pathologies over well-being. How do we expect that future physicians focus on health when they treat their patients, not only in their ill-being, if we do not teach them about well-being?

Based on all of the above, I believe that incorporating aspects of positive psychology into medical education would not only bring us closer to teaching about health as conceived by the WHO, but it could also be a great opportunity to reduce alarming rates of mental health problems that affects medical students worldwide, improve their well-being, and that could even improve learning outcomes. I believe that more attention should be paid to the advances in this field and the possible contributions that can be delivered to medical education, as they could help us to refocus our teaching practices to the ultimate goal of life, happiness.

## Take Home Messages

• Medical students are a population with high vulnerability to mental disorders
• Mental disorders can compromise the health of students, influence their learning processes and patient care
• Not only medical students, but also institutions have a role in student well-being
• Positive psychology and positive education can improve well-being and learning outcomes in medical students
• Positive medical education could be an educational approach that improves medical education

## Notes On Contributors

Alvaro Tala, MD, is a Psychiatrist and Assistant Professor in the Psychiatric University Clinic, Faculty of Medicine, University of Chile, Santiago, Chile.

## References

[ref1] Diener E. Pressman S.D. Hunter J. Delgadillo-Chase D. (2017) If, Why, and When Subjective Well-Being Influences Health, and Future Needed Research’, Applied Psychology: Health and Well-Being. 10.1111/aphw.12090 28707767

[ref2] Myers D. G. and Diener E. (2018) The Scientific Pursuit of Happiness’, Perspectives on Psychological Science. 10.1177/1745691618765171 29592651

[ref3] Rotenstein L.S. Ramos M.A. Torre M. Segal J.B. (2016) Prevalence of Depression, Depressive Symptoms, and Suicidal Ideation Among Medical Students. JAMA. 10.1001/jama.2016.17324 PMC5613659 27923088

[ref4] Seligman M. E. P. Ernst R.M. Gillham J. Reivich K. (2009) Positive education: Positive psychology and classroom interventions’, Oxford Review of Education. 10.1080/03054980902934563

