# Positive medical education: Are we focusing on the right things while teaching?

**DOI:** 10.15694/mep.2018.0000156.2

**Published:** 2018-09-06

**Authors:** Alvaro Tala

**Affiliations:** 1 Universidad de Chile

**Keywords:** Positive Psychology, Positive Education, Medical Education, Well-being

## Abstract

This article was migrated. The article was marked as recommended.

Neuropsychiatric disorders are a global problem and medical students are a population with high vulnerability to mental disorders. Medical education is currently in crisis considering the alarming rates of mental health problems reported in medical students. These problems not only compromise the health of students but also their learning processes and patient care. In this context, it is necessary to move towards a medical education that considers, in addition to the academic performance, the well-being of medical students as a central focus. Positive psychology with interventions at individual level and positive education with interventions at institutional level could provide a response based on the evidence to this problem through positive medical education. This article addressed a personal view of how positive medical education can benefit medical students.

## Perspective

Medical education and well-being have a close relationship. How the teaching and learning processes are developed may affect the well-being of the students ( Moir *et al.*, 2018). There is compelling evidence associating elements of well-being with better outcomes in physical health, mental health, learning, and labor productivity, possibly through mechanisms, such as increased creativity, cognitive flexibility, holistic thinking, resilience, intrinsic motivation, among others ( Seligman *et al.*, 2009; Diener *et al.*, 2017; Myers and Diener, 2018). On the other side, mental health problems not only compromise the health of students but also their learning processes and patient care( Hope and Henderson, 2014; Pacheco *et al.*, 2017).

Medical students are a population with greater vulnerability to mental disorders compared to populations of similar age range due to various factors, such as role transition, sleep deprivation, poor support systems, work overload, academic demands and pressures of the clinical environment ( Goldman, Shah and Bernstein, 2015; Moir *et al.*, 2018). Several studies have shown alarming rates of depressive symptoms up to almost 30% and suicidal ideation up to 11% in this population ( Hope and Henderson, 2014; Goldman, Shah and Bernstein, 2015; Rotenstein *et al.*, 2016) and given how the learning environments are structured nowadays medical students tends to neglect their well-being to prioritize their academic performance. Although universities have implemented various strategies, including mental health programs, mind-body skills programs, changes in curriculum structure, advising/mentoring programs, mindfulness-based stress reduction, among others strategies to promote the well-being of its students from different approaches, they have not been able to find a definitive answer to this problem and even many times are being confronted with that students do not value these strategies as useful for their well-being ( Wasson *et al.*, 2016; Ayala *et al.*, 2017).

In this context, I think the work carried out by positive psychology in the topic of well-being and education stands out. Positive psychology is a field that focuses on the scientific study of the conditions and processes that contribute to the flourishing or optimal functioning of people, groups, and institutions. This field includes the empirical study of well-being from different perspectives, such as gratitude, engagement, forgiveness, flow, optimism, post-traumatic growth, resilience, strengths, emotional regulation, creativity, among others ( Gable and Haidt, 2005; Jeste *et al.*, 2015). I think interventions from the positive psychology point of view could bring benefits to medical education. Proposing interventions to medical students to promote their well-being from the perspective of specific mental disorders may lead to problems, such as the pathologization of normal experiences and focus the strategies on some disorders over others. Also, students may not perceive themselves as sick, or even if they do, the may not seek help in suitable time because of the stigma associated with mental disorders ( Arango *et al.*, 2018; Moir *et al.*, 2018). From the framework of positive psychology, interventions have been studied that could not only reduce depressive symptomatology but also increase well-being in its various components, which with small to moderate effect sizes that could be sustained over time (Sin and Lyubomirsky, 2009; Bolier *et al.*, 2013; Quoidbach, Mikolajczak and Gross, 2015). Some of these interventions also have the advantage of being able to be widely spread online and self-applied without the need of the specific mental health disorder diagnosis. For example, gratitude visit is an intervention that can be taught online and self-appliedwhich consists in writing and delivering a letter of gratitude in person to someone one wants to thank. I think all of these advantages could avoid the issues described above.

Under the umbrella of the positive psychology also positive education exists. Positive education implies the application of principles of positive psychology to education, blending the measurement and development of competencies related to well-being with the development of traditional competencies in education, for example, by helping students to identify their signature character strengths and increasing the use of these strengths in day-to-day life, including learning activities ( Seligman *et al.*, 2009). Within this approach, many great scientists in various fields (e.g. psychology and education) have managed to carry out initiatives worldwide, in countries like the United Kingdom, Australia, Mexico and the United States of America, both in schools and universities, to generate concrete educational programs with measurable results ( Seligman *et al.*, 2009; White and Waters, 2015; Chen, 2016; World Government Summit and IPEN, 2017). This on the basis that well-being would have aspects that could be intervened and strengthened through education.

Unlike positive interventions that can be applied and evaluated at an individual level and sometimes even applied independently of the existing curriculum, curricular or institutional changes are complex processes that are usually shaped by economic, social and cultural aspects. Here is where positive education is needed. Interventions at school level have shown that they could prevent adverse outcomes in mental health and that they can increase good relationships, engagement in learning, cooperation, assertiveness, and self-control, among other qualities ( Seligman *et al.*, 2009; Adler, 2016). I think these qualities can benefit medical students to meet the needs of the modern healthcare.However, there is a lack of data related to positive education in medical curricula across the world. An example of how positive education can be applied in the universities is found in the experience of Tecmilenio University in Mexico. They added to their curriculum a positive psychology course and a well-being based learning ecosystem. They found out that students exposed to this approach outperform students with no-exposure in well-being and learning outcomes. Other changes that can be made at institutional level includechanges in the evaluation system from one based on grades to one consisting on passing or failing, changes in teaching methodologies,such as the use of problem-based learning, reduction of the academic load, increase of the elective courses, establishment of learning communities and implementation of mentoring( Slavin, Schindler and Chibnall, 2014).

I think that more important than the specific interventions, which still require further studies to ensure their effectiveness, is the contribution that the paradigm of positive psychology offers to medical education in the following question: “What is the purpose of teaching/learning?” From my point of view, the potential answer to this question could be that we learn and teach to maximize the well-being of individuals and eventually of the society. In this context, educational institutions are a fundamental place from which well-being can be worked since they are environments that frequently affect the well-being of the students and since they are the place where students spend most of their time. Although WHO defines health as a state of complete physical, mental and social well-being and not merely the absence of disease or infirmity ( *Constitution of WHO: principles*, 2018), in my experience from medical education, the time students spend in medical education tends to focus particularly on disorders and pathologies over well-being. How do we expect that future physicians focus on health when they treat their patients and not only in their ill-being if we do not teach them about well-being?

Based on the discussion above, I believe that incorporating aspects of positive psychology into medical education could bring us closer to teaching about health as conceived by the WHO. From my perspective, it could also be a golden opportunity to reduce the alarming rates of mental health problems that affect medical students worldwide, improve their well-being, and even improve their learning outcomes. I think medical education has focused for a long time in academic outcomes and in the process has compromised the well-being of our students. This could change if we start to measure and promote well-being in medical schools and I believe this can be done by using positive psychology and positive education as frameworks to develop a positive medical education. More attention should be paid to the advances in these fields and the possible contributions that they can deliver to medical education as they could help us to refocus our teaching practices to the ultimate goal of life, happiness.

## Take Home Messages

• Medical students are a population with high vulnerability to mental disorders
• Mental disorders may compromise the health of students, affect their learning processes and patient care
• Not only medical students but also institutions have a crucial role in student well-being
• Positive psychology and positive education can improve well-being and learning outcomes in medical students
• Positive medical education could be an educational approach that improves medical education

## Notes On Contributors

Alvaro Tala, MD, is a Psychiatrist and Assistant Professor in the Psychiatric University Clinic, Faculty of Medicine, University of Chile, Santiago, Chile.

## References

[ref1] Adler A. (2016) Teaching well-being increases academic performance: Evidence from Bhutan, Mexico, and Peru. ProQuest Dissertations and Theses.

[ref2] Arango C. (2018) Preventive strategies for mental health. The Lancet Psychiatry. 10.1016/S2215-0366(18)30057-9 29773478

[ref3] Ayala E. E. (2017) What Do Medical Students Do for Self-Care? A Student-Centered Approach to Well-Being. Teaching and Learning in Medicine. 10.1080/10401334.2016.1271334 28632007

[ref4] Chen C. (2016) The Role of Resilience and Coping Styles in Subjective Well-Being Among Chinese University Students. Asia-Pacific Education Researcher. 10.1007/s40299-016-0274-5

[ref5] Diener E. (2017) If, Why, and When Subjective Well-Being Influences Health, and Future Needed Research. Applied Psychology: Health and Well-Being. 10.1111/aphw.12090 28707767

[ref6] Gable S. L. and Haidt J. (2005) What (and why) is positive psychology? Review of General Psychology. 10.1037/1089-2680.9.2.103

[ref7] Goldman M. L. Shah R. N. and Bernstein C. A. (2015) Depression and suicide among physician trainees: Recommendations for a national response. JAMA Psychiatry. 10.1001/jamapsychiatry.2014.3050 25738529

[ref8] Hope V. and Henderson M. (2014) Medical student depression, anxiety and distress outside North America: a systematic review. Medical Education. 10.1111/medu.12512 25200017

[ref9] Jeste D. V. (2015) Positive psychiatry: Its time has come. Journal of Clinical Psychiatry. 10.4088/JCP.14nr09599 PMC5748141 26132670

[ref10] Moir F. (2018) Depression in medical students: current insights. Advances in Medical Education and Practice. 10.2147/AMEP.S137384 PMC5944463 29765261

[ref11] Myers D. G. and Diener E. (2018) The Scientific Pursuit of Happiness. Perspectives on Psychological Science. 10.1177/1745691618765171 29592651

[ref12] Pacheco J. P. (2017) Mental health problems among medical students in Brazil: a systematic review and meta-analysis. Revista Brasileira de Psiquiatria. 10.1590/1516-4446-2017-2223 PMC7111407 28876408

[ref13] Rotenstein L. S. (2016) Prevalence of Depression, Depressive Symptoms, and Suicidal Ideation Among Medical Students. JAMA. 10.1001/jama.2016.17324 PMC5613659 27923088

[ref14] Seligman M. E. P. (2009) Positive education: Positive psychology and classroom interventions. Oxford Review of Education. 10.1080/03054980902934563

[ref15] Slavin S. J. Schindler D. L. and Chibnall J. T. (2014) Medical student mental health 3.0: Improving student wellness through curricular changes. Academic Medicine. 10.1097/ACM.0000000000000166 PMC4885556 24556765

[ref16] Wasson L. T. (2016) Association Between Learning Environment Interventions and Medical Student Well-being. JAMA. 10.1001/jama.2016.17573 PMC5240821 27923091

[ref17] White M. A. and Waters L. E. (2015) A case study of “The Good School:” Examples of the use of Peterson’s strengths-based approach with students. Journal of Positive Psychology. 10.1080/17439760.2014.920408 PMC4241650 25431614

[ref18] World Government Summit and IPEN (2017) The state of positive education. The state of positive education.

