# Supplementary material for: Positive medical education: Are we focusing on the right things while teaching?
Source: MedEdPublish (2016). 2018 Sep 6;7:156. Originally published 2018 Jul 26. [Version 2] doi: 10.15694/mep.2018.0000156.2 (PMC10701801; doi:10.15694/mep.2018.0000156.2)
Supplement: Peer review history

# Reviewer report: Reviewer response for version 2

**DOI:** 10.21956/mep.20227.r31333

**Published:** 2019-01-02

**Authors:** Hassaan Waqar

**Affiliations:** 1 St Helens and Knowsley Teaching Hospitals NHS Trust – Lead Employer

This review has been migrated. The reviewer awarded 3 stars out of 5 An interesting perspective piece on the effect of mental health on medical students and how as teachers we should take account of this.The abstract succinctly describes how mental health problems amongst medical students can impact their learning and the interventions that can take place to reduce this impact. In the main body of the article, the author details the extent to which mental health problems can impact medical students. The author also explores interventions that have been used by institutions to help students manage these problems. The author focusses on positive psychology as the intervention of choice, however does not explain why this approach is better than the other approaches. Additionally, whilst the importance of positive psychology and later positive education is highlighted, the obstacles to adopting these concepts on an institutional level are not explored. By using a wholistic definition of health, as advocated by the World Health Organisation, the author reinforces the idea of using positive education methods to improve the overall educational experience. This is an excellent idea as it emphasises a wholistic approach to medical education. The positive wholistic approach identified by the author is an area that may require further research in order to identify methods which can be utilised to alter medical education frameworks to shift the focus to wellbeing in medical schools. The take home messages are clear and simple to understand and neatly summarise the main points of the article.Overall, a very interesting article exploring novel methods of improving the quality of medical education by focussing on the wellbeing of medical students.

Reviewer Expertise:

NA

No decision status is available

# Reviewer report: Reviewer response for version 2

**DOI:** 10.21956/mep.20227.r31334

**Published:** 2018-10-03

**Authors:** Sateesh Babu Arja

**Affiliations:** 1 Avalon University School of Medicine

This review has been migrated. The reviewer awarded 4 stars out of 5 I would like to congratulate the author for taking up such a challenging issue in medical education wellness of medical students and positive education. I thoroughly enjoyed reading this paper. It is clearly written and easily understandable. The author revised the paper based on suggestions of the reviewers of previous version. Author clearly explained the interventions and initiatives could be taken to promote wellness of medical students and positive medical education. This paper is useful for all academic administrators who are involved in student affairs and student services and responsible for wellbeing of medical students.

Reviewer Expertise:

NA

No decision status is available

# Reviewer report: Reviewer response for version 2

**DOI:** 10.21956/mep.20227.r31332

**Published:** 2018-09-06

**Authors:** Subha Ramani

**Affiliations:** 1 Harvard Medical School, Brigham and Women's Hospital

This review has been migrated. The reviewer awarded 4 stars out of 5 I enjoyed reading this paper. Positive psychology is a new concept to me and I found it interesting in how the author linked these principles and described its relevance to positive learning. Wellness is a subject that is actively being addressed in medical education, this paper highlights the urgency of wellness interventions for students. The author also clearly describes what interventions could be consistent with the principles of positive learning and emphasizes the responsibility of institutions in this area.The author has responded very well to reviewer comments on the previous version. There is simplicity and clarity in this paper and sentences are shorter and much better written. I recommend this paper to all educators implementing wellness initiatives, all educators who should be monitoring signs of mental disorders among their students and seeking help.

Reviewer Expertise:

NA

No decision status is available

# Reviewer report: Reviewer response for version 1

**DOI:** 10.21956/mep.19114.r27570

**Published:** 2018-08-08

**Authors:** Meghana Sudhir

**Affiliations:** 1 Mohammed Bin Rashid University of Medicine and Health Sciences

This review has been migrated. The reviewer awarded 2 stars out of 5 I do acknowledge the global issue of stress and burn out and its impact on mental health among medical students. I am unable to relate the title and personal view of the author. Personal view or opinion can be more effectively emphasized with more supportive evidence which is lacking in this paper. The lengthy sentences and lack of punctuation takes away the essence of the article. I would suggest the importance of proof reading before submitting. The terms Positive medical education & positive psychology needs more clarity.

Reviewer Expertise:

NA

No decision status is available

# Reviewer report: Reviewer response for version 1

**DOI:** 10.21956/mep.19114.r27573

**Published:** 2018-08-06

**Authors:** Susan Van Schalkwyk

**Affiliations:** 1 Stellenbosch University

This review has been migrated. The reviewer awarded 3 stars out of 5 There can be no doubt that the mental health well-being of students in the health professions is a growing source of concern and requires our attention. The author raises a number of important issues in this regard and the recommendation that a more positive and focused approach to teaching would have value is an interesting one. Further exploration of what is meant by this and how it could manifest in a practical way, would enhance understanding. The title is possibly a little too cryptic. The lengthy sentences also detract from the readability of the piece. In addition, providing some indication of the author's own credentials would provide a context within which the recommendations can be considered.

Reviewer Expertise:

NA

No decision status is available

# Reviewer report: Reviewer response for version 1

**DOI:** 10.21956/mep.19114.r27572

**Published:** 2018-08-03

**Authors:** P Ravi Shankar

**Affiliations:** 1 American International Medical University

This review has been migrated. The reviewer awarded 3 stars out of 5 The title of the paper is interesting. However, I am not sure that the paper fully justifies the title. The author briefly mentions about the importance of positive education in medical schools. One of the challenges for readers like me is that positive education and the topics which should be addressed are not described. I must admit that I am not aware of what exactly is meant by positive education. I would be grateful if the author can explain this in the manuscript. The article can benefit by being more specific and focused on what areas should be covered under positive education. I can sympathize with the author whose first language is not English but the standard of written English could be improved. There are also a few spelling and punctuation errors which can be corrected.

Reviewer Expertise:

NA

No decision status is available

# Reviewer report: Reviewer response for version 1

**DOI:** 10.21956/mep.19114.r27569

**Published:** 2018-07-28

**Authors:** Neslihan Onder-Ozdemir

**Affiliations:** 1 The University of Sheffield

This review has been migrated. The reviewer awarded 5 stars out of 5 Dear Alvaro Tala,Thank you very much for highlighting the ‘significance of neuropsychiatric disorders’ as mental health problems with potentially harmful effects on medical students’ education and patients’ care. Unfortunately, in every stage of education mobbing and bullying are at an alarming stage that are highly likely to bring about devastating effects, including neuropsychiatric disorders. Indeed, fulfilling lives should be on the agenda for the enhancement of medical students’ years in medical education. Indeed, one of your key messages “Positive medical education could be an educational approach that improves medical education” is a concise message. Below are my basic comments about punctuation you may consider:In the Abstract, a “dot” is needed for the last sentence: This article addresses a personal view of how this approach can benefit medical students [.]Please delete the comma after “medical students” in the following sentence:Not only medical students[,] but also institutions have a role in student well-beingPlease delete the “dot” after the question mark in the following sentence:How do we expect that future physicians focus on health when they treat their patients, not only in their ill-being, if we do not teach them about well-being?[.]

Reviewer Expertise:

NA

No decision status is available

# Reviewer report: Reviewer response for version 1

**DOI:** 10.21956/mep.19114.r27571

**Published:** 2018-07-27

**Authors:** Sateesh Babu Arja

**Affiliations:** 1 Avalon University School of Medicine

This review has been migrated. The reviewer awarded 3 stars out of 5 I enjoyed while reading this paper.This is such an interesting topic in the current medical education. Physical and mental well-being of medical students and burnout issues among medical students and residents became hot topics in the medical fraternity. Even recently when I attend the conference of International Association of Medical Sciences Educators (IAMSE), there was a lot of discussion regarding well-being of medical students. It emphasizes the importance of having effective learners support system in all universities. This leaners support systems should be able to provide support in academic and professional development and provide personal support in coping up with the physical and emotional demands of the school. It is good to see authors picked up an important topic. Most of us may like their suggestion to incorporate positive psychology into medical education and curriculum. But I would like to see how this can be incorporated into curriculum. Authors might would like to elaborate on this.

Reviewer Expertise:

NA

No decision status is available
